# Counting Crowds with Perspective Distortion Correction via Adaptive Learning

**DOI:** 10.3390/s20133781

**Published:** 2020-07-06

**Authors:** Yixuan Sun, Jian Jin, Xingjiao Wu, Tianlong Ma, Jing Yang

**Affiliations:** School of Computer Science and Technology, East China Normal University, Shanghai 200062, China; 10175102160@stu.ecnu.edu.cn (Y.S.); 52184506007@stu.ecnu.edu.cn (X.W.); tlma@cs.ecnu.edu.cn (T.M.); jyang@cs.ecnu.edu.cn (J.Y.)

**Keywords:** crowd counting, localization, adaptive learning, convolutional neural network

## Abstract

The goal of crowd counting is to estimate the number of people in the image. Presently, use regression to count people number became a mainstream method. It is worth noting that, with the development of convolutional neural networks (CNN), methods that are based on CNN have become a research hotspot. It is a more interesting topic that how to locate the site of the person in the image than simply predicting the number of people in the image. The perspective transformation present is still a challenge, because perspective distortion will cause differences in the size of the crowd in the image. To devote perspective distortion and locate the site of the person more accuracy, we design a novel framework named Adaptive Learning Network (CAL). We use the VGG as the backbone. After each pooling layer is output, we collect the 1/2, 1/4, 1/8, and 1/16 features of the original image and combine them with the weights learned by an adaptive learning branch. The object of our adaptive learning branch is each image in the datasets. By combining the output features of different sizes of each image, the challenge of drastic changes in the size of the image crowd due to perspective transformation is reduced. We conducted experiments on four population counting data sets (i.e., ShanghaiTech Part A, ShanghaiTech Part B, UCF_CC_50 and UCF-QNRF), and the results show that our model has a good performance.

## 1. Introduction

The goal of the crowd counting task is to count the number of people in an image. The crowd counting task plays an important role in the production, life, disaster management, security monitoring, and public space design [1,2,3]. With the improvement of people’s safety awareness, crowd counting has been paid increasing attention. Recently the crowd counting task has utilized convolutional neural network (CNN) to address the scale variation issue and has achieved good improvements in crowd density estimation [4,5].

However, the perspective distortion of the image is still an important challenge for crowd counting, more specifically, the model is not particularly accurate in predicting avatars with large differences in size in the same image. Hence, how to better handle objects of different sizes is a key to improve the crowd counting model. Recently, the demand for crowd counting is no longer simply counting the total number of people in the image, but also want to locate a specific personal location, so that accurate counting can be performed more accurately. Most of the current work uses Visual Geometry Group (VGG) [6] as a backbone. Subsequently, separately extract different sizes of features after each max pooling operation, and decoded these features. We will obtain the features that size is 1/2, 1/4, 1/8, and 1/16 of the original image size after each max pooling.

The current method is to simply superimpose features of different sizes without considering the combination of different sizes brought by different image inputs and different scene inputs. The degree of perspective transformation in each image is not the same, that is, if our branch information is merged according to the same pattern, then the learned knowledge cannot cover all samples. On this basis, we envisage using a dynamic mechanism to combine branch information according to different image features to achieve the goal of dynamic evolution. Inspired by the adaptive scenario discovery framework (ASD) [7] model, we also propose a dynamic learning branch combination method. Different from ASD, our model is not only concerned with simple counting tasks, but we also add the positioning of specific objects to the model. At the same time, ASD distinguishes between sparse and dense scenes, and our model is to explore the degree of perspective change in the image. In this paper, we propose an adaptive learning framework (CAL) with perspective distortion correction for crowd counting and localization. We employ several of VGG-16 convolution layers for crowd feature extraction before the multiple receptive fields instead of utilizing them directly. Additionally, for exploring the degree of perspective change in the image, four parallel pathways with the counting and localization network named main, scale, middle, and lowest are proposed. The four pathways are designed for the people with different scale, respectively. Besides, we also designed a branch to learn the degree of perspective change. Afterwards, combine the perspective into the output branch of the model.

Our contributions are listed, as follows.

- We propose a novel adaptive framework with perspective distortion correction for crowd counting and localization. Different from the former proposed multiple columns frameworks, we use a branch to dynamically characterize the degree of perspective change of the images. We further verify the effect of our CAL network and compare with the No-CAL methods in order to explain the improvement of our architecture.
- We design a novel size characterization branch to realize both the crowd counting and the localization task.
- We use VGG [6] for the feature extraction structure and the network constructed by four branches (including the main path), which select output features of different sizes. The perspective change in the image is considered to be a linear combination of our four branches and discrete weights, while the adaptation branch aims to portray a continuous perspective change trend and make corresponding corrections.
- We apply our framework to four congested multi-scene crowd counting datasets (i.e., ShanghaiTech Part A, ShanghaiTech Part B, UCF_CC_50, and UCF-QNRF) and prove that our method outperforms the state-of-the-art methods.

In the remaining part of the paper, we discuss related works of crowd counting and localization in Section 2, describe the backbone, the CAL network architecture and training process in Section 3, verify the proposed framework in both qualitative and quantitative extent in Section 4 and finally conclude our work in Section 5.

## 2. Related Work

We present a survey about the recent works of crowd counting and localization in three parts: (1) traditional crowd counting methods; (2) CNNs for counting; and, (3) CNNs for localization. The earliest researches mostly based on detection frameworks, which were used to detect people and to count the number of pedestrians. However, the occlusion, the extremely dense crowds, and high background clutter limited its development, even though some improvement, such as parts-based or shape-based detectors, were proposed. To devote these issues, some researcher proposes the regression-based methods (mapping the features extracted from local images and their counts) took the place of detection-based methods. Since the CNN was proposed, it has been successfully used in various computer vision tasks, which inspired the use of CNN based methods in crowd counting tasks. Though the approaches of crowd counting scenes gained satisfying performance, in several scenes some more detailed information, such as the distribution and the location of the objects, were needed. As a result, researchers improved the CNN frameworks and developed a series of CNN based localization models. Nowadays, the localization tasks with CNN based framework are still the hotspots for researchers.

### 2.1. Traditional Crowd Counting Methods

The early detection-based methods [8,9,10,11] rely on the detection style framework that used the slide window to detect people in images. These methods estimating the number in the low-density crowd scenes by detecting the whole body of the pedestrians. However, in high-density situations, heads are ususally the only visible part due to the occlusions. As a further development, the detectors of some body parts (such as head or head-shoulder [12]) detection methods were proposed. On the other hand, regression-based approaches [13,14,15,16,17,18] regressed the density map of crowds and the integration of which is the crowd counting result. These earlier methods [15,16,17,18] mapped global image features or combined local patch features to do counting, which produces approximately counts. When comparing these two methods, regression-based approaches perform well in high-density situations. Additionally, the detection-based methods can usually handle the counting and localization problems simultaneously.

### 2.2. CNNs for Crowd Counting

Recently, CNN based approaches [19,20,21,22] have shown their advantages in learning the crowd image feature mapping and the people/head detection for both crowd counting [23,24,25,26,27,28] and localization [26,27,28,29,30]. The Multi-column Convolutional Neural Network (MCNN) method is evaluated in [19] which contains three columns of different filters to extract feature of heads in different scales. Sam et al. [21] proposed the Switching-CNN and trained each of three columns with a subset of the patches, while a density selector is designed for extracting the structural and functional differences. Li et al. [31] introduce the CSRNet as an approach to concentrate on encoding the deeper features in congested scenes. Besides, Idrees [32] introduced a deep CNN with composition loss method to satisfy counting, density map estimation, and localization. To handle the small/tiny objects that often appear in crowd counting scenes, Basalamah et al. [27] used the scale-aware object proposal generated by perspective information which handled scale variations and makes the model (SD-CNN) able to detect human heads in both low density and high-density crowd images. Onoro et al. [33] using the Hydra-CNN fuses the multi-scale information provided by heads to handle the crowd counting problems with significant variations in the scene. Additionally, Reference [26] introduced the depth information by leveraging RGB-D data to improve the performance of small object detection. In some occasions, like the wild scenes or the congress scenes, the annotation can be costly. As a result, recent researches [34,35,36] aimed at dealing with the lack of labeled data by self-supervised learning [34,36] or the unsupervised learning [35]. In [34], Wang et al. used the GCC dataset to fine-tune a pre-trained crowd counter and proposed a crowd counting method via domain adaptation, which freed the researchers from data annotations. For the unsupervised ways, Reference [35] presented an unsupervised learning method using Grid Winner-Take-all (GWTA) Counting CNN to learn features from unlabeled crowd images.

### 2.3. CNNs for Localization

As the regression-based crowd counting methods are widely used in the counting scenes, the most direct idea is to handle the localization task by sharpening crowd density maps. However, the low accuracy of density map that was argued in most of the prior studies [28] is still an unignorable drawback. An early anomaly detection and localization method [30] introduced normalcy models jointly show the appearance and dynamics of complex congested scenes in which MDTs are learned at multiple scales to handle the problems of empirical evaluation of anomaly detectors on crowded scenes. The further anomaly detection research [37] proposed an unsupervised approach for crowd scene anomaly detection and localization while using the social network model, which outperformed the former ones. To handle the localization and detection task in the noisy foreground, Chen et al. [38] extracted noisy foreground using the person detector and foreground segmentation. Chen et al. [38] also introduced the new framework of EGR and introduced a new metric for both errors in localization and counting. Nowadays, most researches [26,27,28,33] using both the density map and neural network detector for the localization task. Idrees et al. [32] introduced the composition loss to do the counting, density map estimation and localization in congested scenes simultaneously. Reference [27] devises a scale-aware head detector and using the response map to optimize the detector to make the test results more consistent with population distribution. Instead of the scale-awareness, Reference [26] approached the depth information by designed a depth-aware anchor to initialize the anchor and estimated the bounding box sizes of all heads that were utilized as the ground truth to train the RDNet. Additionally, to satisfy the demanding of large-scale RGB-D dataset, Reference [26] also introduced an RGB-D dataset contains 2193 images and 144,512 headcounts named ShanghaiTechRGBD. And Liu et al. [28] proposed the recurrent attentive zooming network to zoom the detected ambiguous image region into high resolution and using the RAZ Net for re-inspection.

The Fully Convolutional Network (FCN) is proposed for the pixel-level classification of images. Matan et al. [39] extended the classic LeNet [40] to recognize strings of digits. Additionally, in the segmentation of *C. elegans* tissues scene, Ning et al. [41] used the fully convolutional inference to design a convent. In recent years, multi-layered nets have also exploited the fully convolutional computation (such as sliding window detection [42] and image restoration [43]). Moreover, He et al. [44] generated a localized and fixed-length feature with proposals and spatial pyramid pooling. However, the drawback of this hybrid model is that it cannot be learned end-to-end. Taking these case studies into account, Long et al. [45] proposed a fully convolutional network trained end to end and pixel to pixels. The research is known as the first work trained the FCNs end to end for pixel wised segmentation and used the supervised pre-training. Additionally, [46] designed a U-net model, which used a fully-convolutional neural network as the core of the model. The model required the full per-pixel instance segmentation labels for training. Extending from [45], Issam et al. [47] designed a novel loss function for the FCN model, called localization-based counting loss (LC), and named this new FCN detection-based model with LC function the LC-FCN. Nowadays, Sam et al. [48] proposed the LSC-CNN model while using the multi-column architecture to fulfill the reliably head detection, automatic head size estimation and high precision crowd counting features simultaneously. As a multi-column FCN-liked architecture, the model performs ideally across sparse to dense crowds and only requires the point annotation.

As we said at the beginning of this section, the crowd counting technology has been well developed in recent years. At the initial stage of crowd counting, researchers used sliding windows and regression methods to obtain the number of people in the image. However, with the deepening of research problems and the increase in the number of people in the image, the traditional crowd counting technology has been unable to meet those issues. As such, researchers began to explore CNN-based methods. Presently, the CNN-based method can achieve good results, but the traditional CNN-based method can only get the prediction number, and cannot calibrate the localization of the crowd in the image, so the localization method is proposed. At present, most of the localization task base on FCN structure and, at the same time, the image features of different sizes are stitched and decoded. However, this method is not ideal for scenes with a severe perspective change. Therefore, based on FCN, we propose an adaptive learning framework with perspective distortion correction for crowd counting and localization. We achieve an end-to-end regression method using CNNs, which takes the entire image as input and obtain greater accuracy when compared to previous approaches.

## 3. Framework

**Figure 1:**
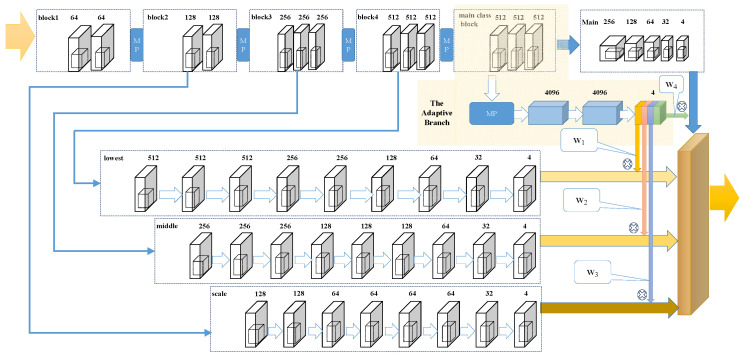
The architecture and weight of CAL.

We propose a novel model that contains three parts: the backbone, the pathways, and the adaptive branch. We design a novel framework named Adaptive Learning Network (CAL) and the architecture is shown in Figure 1. In the following parts, we introduce the structure and implementation in detail.

### 3.1. Backbone

Presently, the mainstream method for extracting features from the crowd counting task is to use the VGG [6] network as a backbone. The backbone network utilizing can be separated into two ways: starting from scratch to designing a new network (e.g., [19]) or migrating a pre-trained subnet from an existing network (e.g., [31,47,49]). Between these two categories, the second way have more advantages in both time-saving and efficiency. Our network design also follows this principle. We first designed a feature extraction structure with VGG16 as the backbone. However, we duplicated and fine-tuned some blocks to adapt the feature extraction task with multiple resolutions. More specifically, our backbone removes the fully connected layer of VGG16, as shown in Table 1. Besides, our VGG model first uses the ImageNet dataset [50] for pre-training.

### 3.2. The Pathways

Following the general principles of localization network design, our network design also uses the FCN structure. Similar to many networks, we also set up four different branches to decode 1/2, 1/4, 1/8, and 1/16 of the original image size, four parallel pathways with the counting, and localization network named main, scale, middle, and lowest are proposed. Table 2 shows the pathways configuration.

### 3.3. The Adaptive Branch

However, unlike other networks connected directly in series, we propose using different weights to combine the output of each branch. We constructed a self-learning classification branch, named the adaptive branch. The input of this branch is the feature parameter extracted by VGG16. The branch structure is as follows: conv (3, 512, 1)–conv (3, 512, 1)–conv (3, 512, 1)–pool(2)–FC (25088, 4096)–RELU–FC(4096, 4096)–RELU–FC (4096, 4). Where ‘conv’ represents a convolutional layer, and ‘pool’ represents a max-pooling layer, ‘FC’ represents the fully connected layer, ‘RELU’ represents Rectified Linear Unit. The numbers in the parentheses are respectively kernel size, the number of channels and dilation rate. Finally, we can obtain four channels (CH) and then normalize each channel separately after summing. The weight coefficient is obtained, as shown in Equation (1).

We provide different weights through the adaptive branch, in order to determine the proportion of the original image that scales different sizes in the result. Using different weight values determines which size image details we will pay more attention to. If we pay attention to more than 1/16 of the image, then it is bound to be ignored for those particularly small heads. Conversely, if we pay attention to 1/2 of the image, then we are bound to pay more attention to those larger heads. Through dynamic learning, we can allocate the proportion of images with different degrees of attention according to the specific scene. That helps eliminate the effects of perspective changes.

(1) $$w_{i}=\frac{\left|\sum CH_{i}\right|}{\sum_{i=1}^{4}\left|\sum CH_{i}\right|+10^{-9}}\left(i=1,2,3,4\right)$$

### 3.4. Implementation Details

Our perspective distortion correction model is implemented using PyTorch [51]. To train the model, we first initialize the batch size as typically four, while the momentum parameter is set as 0.9. We then set the learning rate of 1$e^{-3}$ for all the datasets as initial, and use SGD [52] for training. For the training of UCF_CC_50, we especially use the five-fold cross-validation to make full use of the datasets to test the effectiveness of the algorithm.

#### 3.4.1. Loss Function

Following the design of loss function in [4,5], we proposed the loss function as Equation (2). In which N, $X_{i}$, and θ represent for the batch size, the *i*th input image, and a set of trainable parameters, respectively. Besides, $\gamma_{i}$ is the ground truth of $X_{i}$. Additionally, $Y(X_{i};\theta )$ stands for the estimated density map generated by our proposed model with parameters θ. L(θ) denotes the loss function between the estimated results and the ground truth.

(2) $$L(\theta )=\frac{1}{2N}\sum_{n=1}^{N}\left(∥\gamma \left(X_{i};\theta \right)-\gamma_{i}^{GT}{∥}_{2}^{2}\right)$$

#### 3.4.2. Density Map Generation

CNN needs to process continuous data for crowd counting tasks. As a result, we have to convert the discrete point annotated data (including the annotation of ground truth and the result of prediction) into the density map. The conversion is pixel level and the idea is to convert the point annotation information into images that probably contain density information. The details of the operation are shown in Algorithm 1.
**Algorithm 1:** Ground-truth generation
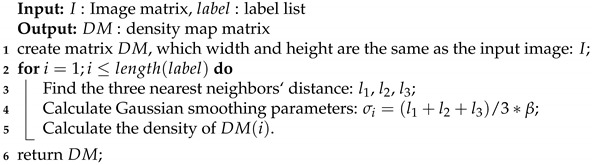


## 4. Experiments

In this section, we introduce three popular crowd counting datasets that are frequently used in crowd counting and localization tasks. Besides, several ways to evaluate the performance of the architectures are introduced. Afterwards, we compare the previous experimental results and evaluate our method on these datasets.

### 4.1. Evaluation Metrics

Several ways are used to evaluate both the person detection and counting performance. For the counting evaluation, the commonly used mean absolute error (MAE) and mean square error (MSE) is used by us to measure the deviation of the prediction and the ground truth. The MAE and the MSE are defined as:

(3) $$MAE=\frac{1}{T}\sum_{t=1}^{T}\left|\mu_{t}-G_{t}\right|$$

(4) $$MSE=\frac{1}{T}\sum_{t=1}^{T}{\left(\mu_{t}-G_{t}\right)}^{2}$$

where *T* is the sum amount of testing frames. While $\mu_{t}$ and $G_{t}$ are the frame t prediction count and the ground-truth count of pedestrians, respectively.

### 4.2. Datasets

**Figure 2:**
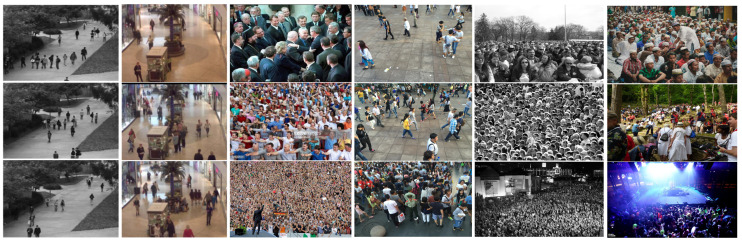
Sample images from various datasets. In order from left to right, each column is in turn UCSD [53], Mall [16], Shanghai Tech PartA [19], Shanghai Tech Part B [19], UCF_CC_50 [17], UCF-QNRF [32]. It is obvious that in UCSD and Mall dataset, the images providing no variation in perspective across images.

Currently, various of public datasets for crowd counting task is available, such as MALL [16], UCSD [53], ShanghaiTech [19], UCF_CC_50 [17], UCF-QNRF [32], etc. The comparison of the images in the listed datasets is shown in Figure 2. In our experiment, we evaluate the proposed model on three crowd counting datasets, including ShanghaiTech [19], UCF_CC_50 [17], and UCF-QNRF [32]. In the latter parts, we present the chosen datasets and explain why these datasets are chosen.

**ShanghaiTech.** Shanghai Tech [19] is one of the largest large-scale datasets in recent years which consists of total 1198 crowd images with 330,165 annotations. The dataset is divided into two sets, named Part A (SHT A) and Part B (SHT B), respectively. Part A is composed of images randomly selected from the Internet, in which the density fluctuates between 33 and 3139 people per image and with an average count of 501.4. In contrast, images in Part B are taken from a busy street of Shanghai and the crowd distribution of which is less diverse and sparser (123.6 in average).

**UCF_CC_50.** UCF_CC_50 [17] is the first challenging dataset on multiple counts created from Web images. The dataset contains various densities and different perspective distortions for multiple scenes. Being a small set of 50 images with crowd counts ranging in 50 to 4543, the dataset poses a serious problem for deep neural networks.

**UCF-QNRF.** UCF-QNRF [32] is collected from Web Search, Flickr, and Hajj footage, which was first introduced by Idrees et al. in [32]. The dataset is consist of a 1201 images train set and a 334 images test set with 1.25 million annotations in total and the density of images varying from 49 people per image to 12,865.

Extracting from these three datasets, the ideal dataset to examine the performance can be concluded as the following list:

- **Challenging images** Some challenging images are necessary to evaluate the performance of the model in extreme conditions. As the development of the crowd counting methods, most of them perform stably in the sparse scenes. As a result, our model focus on improving the performance in congestion crowds and achieve localization tasks. For the crowd counting and localization task, images of some exceeding congestion crowds are the ideal material to evaluate the robustness and the accuracy of our model.
- **Proper density distribution** The distribution of the images can directly affect the performance of the model in the scenes with different levels of congestion. The proper amount of sparse, middle and congested images can improve training accuracy and make verification more effective.
- **Multiple scenes** The dataset contains multiple scenes, such as the street view, the market view, the live show view, etc., can improve the robustness of our model. The multiple scenes is not only the images take from a different location but also the different condition of weather (such as rainy and foggy), light intensity etc., which can affect the performance of our model, especially in the localization task.

In conclusion, the chosen datasets can well meet these issues while the Mall [16] and the UCSD [53] are insufficient in some respects. This explains why we exclude these two datasets.

### 4.3. Results and Discussion

**Figure 3:**
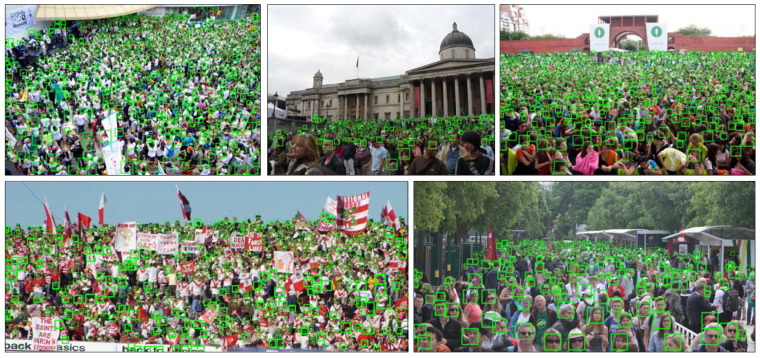
Qualitative results on the ShanghaiTech Part A.

**Figure 4:**
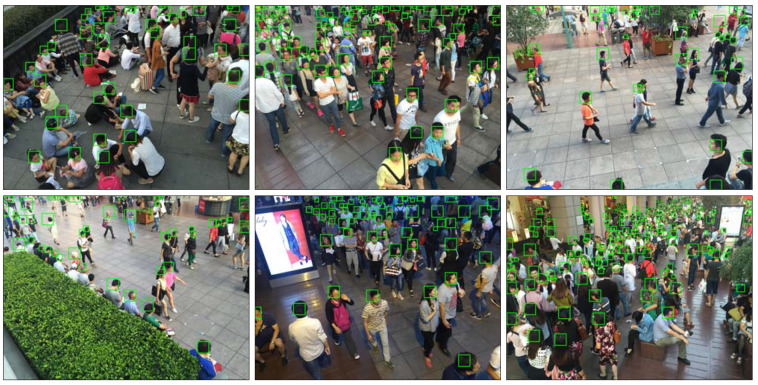
Qualitative results on the ShanghaiTech Part B.

**ShanghaiTech.** Following the introduction of ShanghaiTech dataset above, we evaluated the proposed framework with several state-of-the-art methods, including the localization method utilizing the adaptive fusion scheme named RAZNet [28], the LSC-CNN [48] with different receptive fields and ASD [49] introducing the adaptive scenario discovery framework. Table 3 summarizes the MAE and MSE of the former approaches and ours in two parts of ShanghaiTech. On Part A of ShanghaiTech, we achieve an impressing improvement of 2.1 of absolute MAE value over ASD [49] and 1.6 of MAE over the state-of-the-art RAZNet [28]. When compared with the state-of-the-art (LSC-CNN [48]) on Part B, our CAL network also achieved the best MAE of 8.1 and MSE of 11.9. As the output of our crowd counting and localization model, Figure 3 and Figure 4 show the localization performance of some images from part A and Part B, respectively.

**Figure 5:**
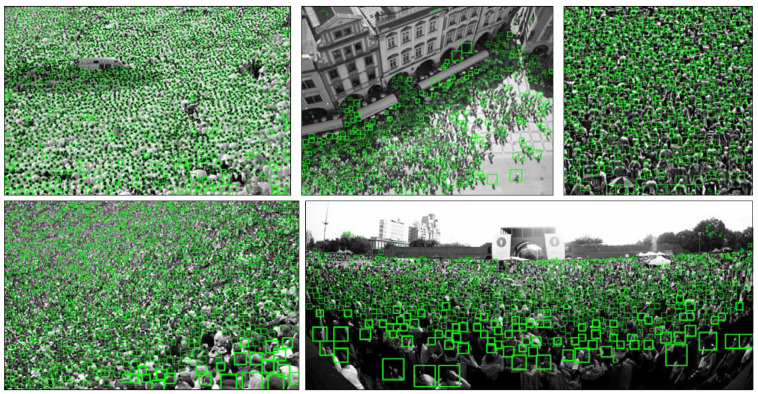
Qualitative results on the UCF_CC_50.

**UCF_CC_50.** As a challenging crowd counting dataset introduced above, we also evaluated the CAL in UCF_CC_50. The results are shown in Table 4 and the instance results are reported in Figure 5. The same as the results on ShanghaiTech, the proposed framework shows better results, and the performance improves on the former state-of-the-art results by 14.2 for the MAE metric, which shows the less volatility of the model in high crowd density images.

**Figure 6:**
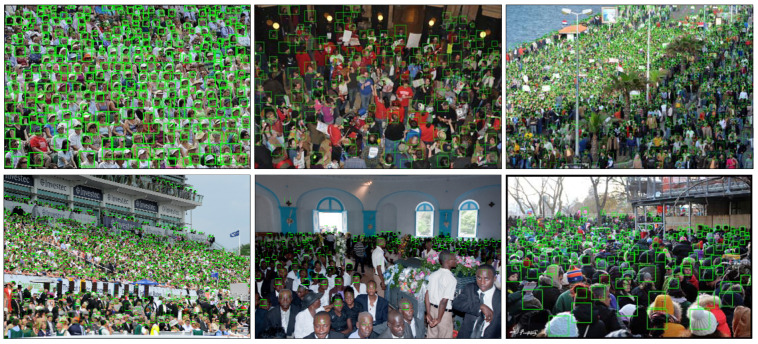
Qualitative results on the UCF-QNRF.

**UCF-QNRF.** Follow the process and the idea of the other two datasets, we use MAE as the evaluation metric and keep the consistent detail for training. Table 5 compares our CAL model with state-of-the-art methods. It is obvious that our model outperforms all of the preceding models. Especially comparing with other localization methods, our network improves at least 10.2 in MAE. Additionally, we provide the performance in predicting the bounding box for localization in Figure 6, which illustrates the localization performance of some images in UCF-QNRF.

### 4.4. Ablation Studies

**Figure 7:**
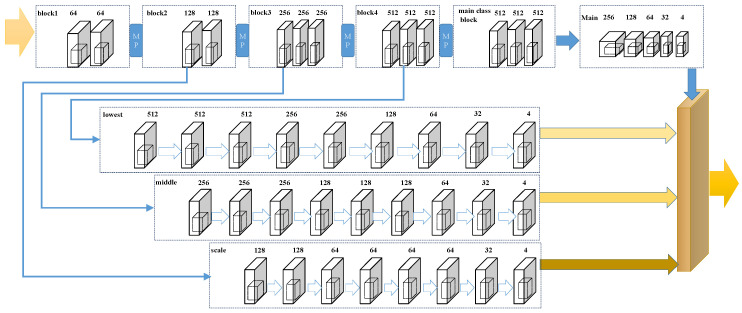
The architecture and weight of NO-CAL.

In this part, we focus on two issues regarding the effectiveness of the structure of the multi-branch network and the performance of the adaptive branch. For this issue, we adjust our model and remove the adaptive branch to make it similar to some normal multi-column models (as shown in Figure 7). Moreover, we name the adjusted model the ‘NO-CAL’. We removed the adaption branch is that we want to explore the improvement effect of the adaption branch on the model. We removed the adaption branch and create the NO-CAL structure in order to better compare the experimental results. To respond to the first issue, we compared our models (CAL & NO-CAL) with the previous multi-branch networks. Additionally, for the second issue, we make a comparison between our CAL model and the NO-CAL model. The results are shown in Table 6 and Table 7.

#### 4.4.1. The Effectiveness of the Multi-Branch Structure

Table 6 shows the comparison of the former multi-branch structure with our design on ShanghaiTech, UCF_CC_50 and UCF-QNRF. It can be seen that our design outperforms the previous methods (MCNN [19], Switch-CNN [21], CMTL [54]). Additionally, the result shows that even the adaptive-branch-cutoff model (NO-CAL), the performance still at least improves on the former results (Part A: 70.8 vs. 90.4; Part B: 14.2 vs. 21.6; UCF_CC_50: 258.9 vs. 318.1; UCF-QNRF: 163.7 vs.228 on MAE). Moreover, the performance is much better than the NO-CAL structure (Part A: 7.3; Part B: 6.1; UCF_CC_50: 47.5; UCF-QNRF: 53.4 improvement on MAE). This is an illustration of the effectiveness of our multi-branch structure.

#### 4.4.2. The Effectiveness of the Adaptive Branch

The previous method cannot handle the perspective distortion challenge properly, as discussed in Section 1. To deep-in validate if our proposed method is affected by the adaptive branch, we first conduct experiments with the CAL model and the same model cancels the adaptive branch (names NO-CAL). We validated both of the models on the three datasets, and the results are revealed in Table 7. It is shown that the CAL model visibly outperforms the NO-CAL, which is due to the good handling of the perspective distortion challenge. The experiment proves the effectiveness of the adaptive branch.

To compare the efficiency of our adaptive branch, we evaluate its time performance. Because the size of the image of the ShanghaiTech Part B is the fix, we use ShanghaiTech Part B as a benchmark to test the time efficiency of the model. The CAL achieves 12 FPS detection speed on an Nvidia TITAN XP GPU and the NO-CAL achieves 13 FPS detection speed on an Nvidia TITAN XP GPU during inference. It may take a little time to use the adaptive branch, but the time spent is in an acceptable range as compared with the improved accuracy.

As our ablation study shows, our design of the network structure is effective and well-performed among three chosen datasets.

## 5. Conclusions

In this paper, we have presented a novel architecture for counting crowds with perspective distortion correction via adaptive learning. The focus of our method is to use a dynamic learning network to learn the dynamic combination relationship under different samples, and use this dynamic combination relationship to form different ratios for each image sample. Experimental comparisons with the state-of-the-art approaches (at most 15 methods) on ShanghaiTech, UCF_CC_50, and UCF-QNRF showed the effectiveness and efficiency of our proposed adaptive scenario discovery framework for the crowd counting task.

## Figures and Tables

**Table 1:** The struct of backbone.

Input (224 × 224 RGB Image)				
	Channels Number	Kernel_Size	Stride	Size
Conv1_1	64	3	1	224 × 224
Conv1_2	64	3	1	224 × 224
Max Pooling	-	2	2	112 × 112
Conv2_1	128	3	1	112 × 112
Conv2_2	128	3	1	112 × 112
Max Pooling	-	2	2	56 × 56
Conv3_1	256	3	1	56 × 56
Conv3_2	256	3	1	56 × 56
Conv3_3	256	3	1	56 × 56
Max Pooling	-	2	2	28 × 28
Conv4_1	512	3	1	28 × 28
Conv4_2	512	3	1	28 × 28
Conv4_3	512	3	1	28 × 28
Max Pooling	-	2	2	14 × 14
Conv5_1	512	3	1	14 × 14
Conv5_2	512	3	1	14 × 14
Conv5_3	512	3	1	14 × 14

**Table 2:** The config of the pathways.

Orgin Image Size: 224 × 224				
Input Size	14 × 14	112 × 112	56 × 56	28 × 28
	Main	Scale	Middle	Lowest
The config of the Pathways	conv(3, 512) relu conv(3, 512) relu conv(3, 512) relu conv(3, 256) relu conv(3, 128) relu conv(3, 64) relu conv(3, 32) relu conv(3, 4) relu	conv(3, 128) relu conv(3, 128) relu conv(3, 64) relu conv(3, 64) relu conv(3, 64) relu conv(3, 64) relu conv(3, 32) relu conv(3, 4) relu	conv(3, 256) relu conv(3, 256) relu conv(3, 256) relu conv(3, 128) relu conv(3, 128) relu conv(3, 128) relu conv(3, 64) relu conv(3, 32) relu conv(3, 4) relu	conv(3, 512) relu conv(3, 512) relu conv(3, 512) relu conv(3, 256) relu conv(3, 256) relu conv(3, 128) relu conv(3, 64) relu conv(3, 32) relu conv(3, 4) relu

**Table 3:** The comparison among the state-of-the-arts and our approach in ShanghaiTech (Part A & Part B). The best result is in bold.

	Methods	Part A		Part B	
		MAE	MSE	MAE	MSE
Counting	MCNN [19]	110.2	173.2	26.4	41.3
	CMTL [54]	101.3	152.4	20	31.1
	TDF-CNN [55]	97.5	145.1	20.7	32.8
	Switching CNN [21]	90.4	135	21.6	33.4
	SaCNN [56]	86.8	139.2	16.2	25.8
	MSCNN [57]	83.8	127.4	17.7	30.2
	ACSCP [58]	75.7	102.7	17.2	27.4
	CP-CNN [59]	73.6	106.4	20.1	30.1
	D-ConvNet-v1 [25]	73.5	112.3	18.7	26
	DRSAN [60]	69.3	**96.4**	11.1	18.2
	CSRNet [31]	68.2	115	10.6	16
	SANet [61]	67	104.5	8.4	13.6
	PACNN [62]	66.3	106.4	8.9	13.5
	ASD [49]	65.6	98	8.5	13.7
Localization	RAZNet [28]	65.1	106.7	8.4	14.1
	RDNet [26]	-	-	8.8	15.3
	LC-FCN8 [47]	-	-	13.14	-
	LSC-CNN [48]	66.4	117	8.1	12.7
	CAL	**63.5**	99.2	**8.1**	**11.9**

**Table 4:** The comparison among the state-of-the-arts and our approach in UCF_CC_50. The best result is in bold.

	Methods	MAE	MSE
Counting	Idrees 2013 [17]	468.0	590.3
	Zhang 2015 [63]	467.0	498.5
	MCNN [19]	377.6	509.1
	MSCNN [57]	363.7	468.4
	TDF-CNN [55]	354.7	491.4
	CMTL [54]	322.8	397.9
	Switching CNN [21]	318.1	439.2
	SaCNN [56]	314.9	424.8
	CP-CNN [59]	298.8	320.9
	PACNN [62]	267.9	357.8
	CSRNet [31]	266.1	397.5
	SPN [64]	259.2	335.9
	SANet [61]	258.4	334.9
	HA-CCN [65]	256.2	348.4
Localization	LSC-CNN [48]	225.6	**302.7**
	CAL	**211.4**	306.7

**Table 5:** The comparison among the state-of-the-arts and our approach in UCF-QNRF. The best result is in bold.

	Method	MAE	MSE
Counting	Idrees 2013 [17]	315	508
	MCNN [19]	277	426
	CMTL [54]	252	514
	Switching CNN [21]	228	445
	HA-CCN [65]	118.1	180.4
	TEDnet [66]	113	188
	RANet [67]	111	190
Localization	RAZNet [28]	116	195
	CL [32]	132	191
	LSC-CNN [48]	120.5	218.2
	CAL	**110.3**	**178.2**

**Table 6:** The comparison between other structure and our approach.

	ShanghaiTech Part A		ShanghaiTech Part B		UCF_CC_50		UCF-QNRF	
	MAE	MSE	MAE	MSE	MAE	MSE	MAE	MSE
MCNN [19]	110.2	173.2	26.4	41.3	377.6	509.1	277	426
CMTL [54]	101.3	152.4	20	31.1	322.8	397.9	252	514
Switching CNN [21]	90.4	135	21.6	33.4	318.1	439.2	228	445
NO-CAL	70.8	119.5	14.2	18.9	258.9	369.0	163.7	200.9
CAL	63.5	99.2	8.1	11.9	211.4	306.7	110.3	178.2

**Table 7:** The comparison between the NO-CAL structure and our approach.

	ShanghaiTech Part A		ShanghaiTech Part B		UCF_CC_50		UCF-QNRF		FPS
	MAE	MSE	MAE	MSE	MAE	MSE	MAE	MSE	ShanghaiTech Part B
CAL	63.5	99.2	8.1	11.9	211.4	306.7	110.3	178.2	12
NO-CAL	70.8	119.5	14.2	18.9	258.9	369.0	163.7	200.9	13
